# Experiments on the Use of Salt

**Published:** 1850-06

**Authors:** 


*Experiments on the Use of Salt.*—M. Plouviez recently presented a memoir to the Academy of Medicine, detailing the results of a series of experiments he has been engaged upon, with the view of determining the part which salt plays in alimentation. To insure accuracy, he had to make choice of persons who led regular lives, continued their habitual mode of alimentation, took the salt at a meal it is not usually taken at, viz.: in the morning, (with milk,) and were weighed before, after, and during the intervals of the experiments. He found more than 25 persons who fulfilled these conditions; but he does not detail the experiments made upon these, as the results only differed in some shades from those observed upon himself. Some of the persons experimented upon, increased in weight from 500 to 2500 grammes in 30 days, and that only from the daily use of from 6 to 10 grammes of salt. Others increased from 5,000 to 10,000 grammes in three or four months. Some acquired more strength and vigour, without any of the inconveniences of excess of nutrition, while others suffered from all the inconveniences of plethora, until the regimen was changed. The nutritive power of the salt was always most observable in feeble, lymphatic subjects. The experiments would at first seem to support the opinion of those who state that 1 lb. of salt will produce 10 lbs. of flesh; but if the regimen is continued from 5 to 10 months or more, the progressive increase of weight is no longer observed, a stationary condition ensuing, the blood being now as rich and nutrition as complete as possible. This fact explains the opposite conclusions arrived at by different observers. The appetite is sometimes found to increase during the first 8 or 10 days, then to resume its normal condition, and after the first or second month to diminish. The most general and certain effect is the increase of the strength; heat is more readily generated, and the exposure to cold better borne.

M. Plouviez's experiments upon himself were commenced in May, 1842, when he weighed 75 kilogrammes. Beginning with a teaspoonful, he increased it to a tablespoonful (from 4 to 6 grammes,) and continued this daily for 4 months. By the end of June his strength and weight (80 kilogrammes,) had both augmented. By the end of July he found himself heavy and oppressed, and this feeling increasing, he was bled the 30th of August—his weight having by this time increased to 85 kilogrammes. During September he suspended the use of the salt, and finding, on resuming it again in October, that his head again became oppressed, he was bled a second time. He made no further experiments on himself during 1843–'45, during which period his weight continued at 83 kilogrammes.

Resuming the experiment again November 1st, 1846, he took until the 2d of December 4 or 5 grammes daily, and from the 3d of December 10 grammes—his weight increasing to 85 kilogrammes. The former symptoms of determination to the head coming on, he was bled on the

28th, and the blood analyzed by M. Poggiale. He then resumed his ordinary regimen for 66 days, lost 2 kilogrammes in weight, and feeling quite well, was again bled, in order that his blood might be again analyzed. The two analyses gave the following results:

| | First Analysis. | Second Analysis. |
|---|---|---|
| Water | 767.60 | 779.92 |
| Globules | 143.00 | 130.08 |
| Fibrine | 2.25 | 2.10 |
| Albumen | 74.00 | 77.44 |
| Fatty matters | 1.31 | 1.13 |
| Salts and extractive | 11.84 | 9.33 |

In the first analysis 6.10 of chloride of soda, and 1.50 oxide of iron were found, and in the second 4.40 chloride of soda and 1.26 oxide of iron.

On the 4th of June, 1847, he resumed the use of the salt, and by the 22d of August had regained his weight of 85 kilogrammes, and was bled in consequence of giddiness. He omitted it, with loss of weight, until the 20th of September, when he resumed it, and reacquired the weight by the 26th of October. He now left off the salt until January, 1848, and then continued it, with intervals of suspension, until February, 1849, the same results being observed. From March to July he suppressed the use of all salt at his meals, and found as the result a loss of weight and a feeling of debility produced.

M. Plouviez in an accompanying note, states that he regards salt: 1. As a condiment until it enters the stomach. 2. As reacting through its bases upon this viscus, and the intestinal canal. 3. As increasing the quantity of chyle by its action on the elements of the chyme. 4. As an excitant of the intestinal absorbents. 5. As a useful modifier of the blood, by diminishing the proportion of its water. 6. As a principal agent in the solution of albumen and fibrine. 7. As one of the agents tending to produce or increase the globules. 8. As a powerful coadjutor in the act of hematosis, without the aid of which the blood does not become reddened in its contact with oxygen. 9. As a valuable auxiliary in the intimate acts of assimilation and deassimilation.

M. Robinet, in reporting upon the memoir reserves his opinion of its scientific value, but does not hesitate to compliment the author for his devotion in having thus undergone a saline regimen during 735 days, spread over 25 months of three different years, leading on several occasions to so much indisposition as to call for blood-letting. *Brit. and For. Med.-Chirurg. Rev.*, Jan., 1850, from *Bulletin de l' Acad. de Med.*, tom. xiv. pp. 1028, 1077–'80.

